# Supplementary material for: Identification of photoperiod-induced specific miRNAs in the adrenal glands of Sunite sheep (Ovis aries)
Source: Front Vet Sci. 2022 Jul 22;9:888207. doi: 10.3389/fvets.2022.888207 (PMC9354845; doi:10.3389/fvets.2022.888207)
Supplement: Supplementary file 1 [file Data_Sheet_1.ZIP › Supplementary file/miRDeep2 file.zip!/miRDeep2 file/Know_Structure/LP42A2/oar-let-7d.pdf]

miRBase precursor : oar-let-7d
Total read count : 18548
oar-let-7d read count : 16267
remaining reads : 2281

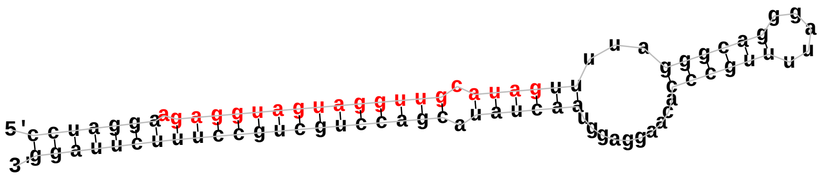

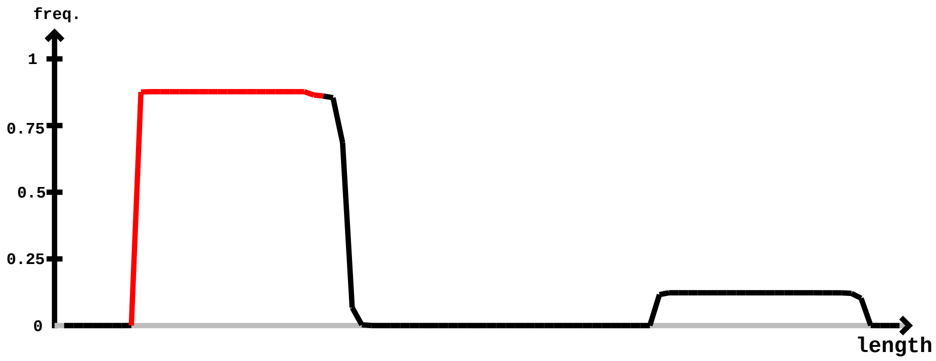

oar-let-7d

```
5'- ccuaggaagagguaguagguugcauaguuuuagggcagggauuuugcccacaaggagguaacuauacgaccugcugccuuucuuagg   -3'    exp
    (((((((.((((((((((((((.((((((...((((((.....))))))..........)))))).)))))))))))))))))))))   reads      mm        sample
    ......aagagguaguagguugcauagu...........................................................   5          0         seq
    ......aagagguaguagguugcauaguu..........................................................   2          0         seq
    .......agagguaguagguugca...............................................................   3          0         seq
    .......agagguaguagguugcau..............................................................   227        0         seq
    .......agagguaguagguugcaua.............................................................   74         0         seq
    .......agagguaguagguugcauag............................................................   116        0         seq
    .......agagguaguagguugcauagu...........................................................   3121       0         seq
    .......agagguaguagguugcauaguu..........................................................   11449      0         seq
    .......agagguaguagguugcauaguuu.........................................................   1194       0         seq
    .......agagguaguagguugcauaguuuu........................................................   50         0         seq
    .......agagguaguagguugcauaguuuua.......................................................   3          0         seq
    ........gagguaguagguugcauagu...........................................................   3          0         seq
    ........gagguaguagguugcauaguu..........................................................   16         0         seq
    ........gagguaguagguugcauaguuu.........................................................   4          0         seq
    .............................................................cuauacgaccugcugcc.........   2          0         seq
    .............................................................cuauacgaccugcugcU.........   1          1         seq
    .............................................................cuauacgaccugcugccu........   1          0         seq
    .............................................................cuauacgaccugcugccuu.......   1          0         seq
    .............................................................cuauacgaccugcugccuuu......   34         0         seq
    .............................................................cuauacgaccugcugccuuuc.....   323        0         seq
    .............................................................cuauacgaccugcugccuuucu....   1787       0         seq
    .............................................................cuauacgaccugcugccuuucuu...   7          0         seq
    ..............................................................uauacgaccugcugccuuu......   5          0         seq
    ..............................................................uauacgaccugcugccuuuc.....   13         0         seq
    ..............................................................uauacgaccugcugccuuucu....   106        0         seq
    ..............................................................uauacgaccugcugccuuucuu...   1          0         seq
```
